# The Potent G-Quadruplex-Binding Compound QN-302 Downregulates S100P Gene Expression in Cells and in an In Vivo Model of Pancreatic Cancer

**DOI:** 10.3390/molecules28062452

**Published:** 2023-03-07

**Authors:** Ahmed A. Ahmed, William Greenhalf, Daniel H. Palmer, Nicole Williams, Jenny Worthington, Tariq Arshad, Shozeb Haider, Effrosyni Alexandrou, Dilek Guneri, Zoe A. E. Waller, Stephen Neidle

**Affiliations:** 1 The School of Pharmacy, University College London, London WC1N 1AX, UK; 2 Guy’s Cancer Centre, Guy’s Hospital, London SE1 9RT, UK; 3 Department of Molecular and Clinical Cancer Medicine, University of Liverpool, Liverpool L69 7BE, UK; 4 AXIS Bioservices, Coleraine BT51 3RP, Northern Ireland, UK; 5 Qualigen Therapeutics, Carlsbad, CA 92011, USA

**Keywords:** pancreatic cancer, quadruplex, biophysics, S100P, transcriptome, MIA PaCa-2 xenograft, molecular modeling

## Abstract

The naphthalene diimide compound QN-302, designed to bind to G-quadruplex DNA sequences within the promoter regions of cancer-related genes, has high anti-proliferative activity in pancreatic cancer cell lines and anti-tumor activity in several experimental models for the disease. We show here that QN-302 also causes downregulation of the expression of the *S100P* gene and the S100P protein in cells and in vivo. This protein is well established as being involved in key proliferation and motility pathways in several human cancers and has been identified as a potential biomarker in pancreatic cancer. The *S100P* gene contains 60 putative quadruplex-forming sequences, one of which is in the promoter region, 48 nucleotides upstream from the transcription start site. We report biophysical and molecular modeling studies showing that this sequence forms a highly stable G-quadruplex in vitro, which is further stabilized by QN-302. We also report transcriptome analyses showing that *S100P* expression is highly upregulated in tissues from human pancreatic cancer tumors, compared to normal pancreas material. The extent of upregulation is dependent on the degree of differentiation of tumor cells, with the most poorly differentiated, from more advanced disease, having the highest level of *S100P* expression. The experimental drug QN-302 is currently in pre-IND development (as of Q1 2023), and its ability to downregulate S100P protein expression supports a role for this protein as a marker of therapeutic response in pancreatic cancer. These results are also consistent with the hypothesis that the *S100P* promoter G-quadruplex is a potential therapeutic target in pancreatic cancer at the transcriptional level for QN-302.

## 1. Introduction

Pancreatic cancer, of which the most common form by far is pancreatic ductal adenocarcinoma (PDAC), is one of the most intractable of all human cancers [1,2,3]. Early stages of the disease are largely asymptomatic, so presentation is commonly at stages 2–4. Surgery is possible in only a small percentage of cases, and therapeutic intervention by single-agent or combination chemotherapy only rarely produces an increase in life expectancy beyond 1–3 years [4]. This dismal overall picture has not significantly changed in 30 years. Even though many small-molecule experimental drugs and immunotherapies have reached the clinical trial stage, remarkably few have had a significantly greater effect on survival than gemcitabine, for many years the (palliative) standard of care/drug of choice in the clinic [5,6,7,8]. To date, no targeted therapy has received clinical approval in PDAC, although the new generation of KRAS G12D inhibitors may have promise [9,10,11,12,13]. Genomic studies of PDAC have demonstrated the complexity and heterogeneity of the disease [14,15,16,17], which are major factors hindering effective precision medicine approaches to treatment [18,19] and the development of clinically useable biomarkers [20].

**Figure 1:**
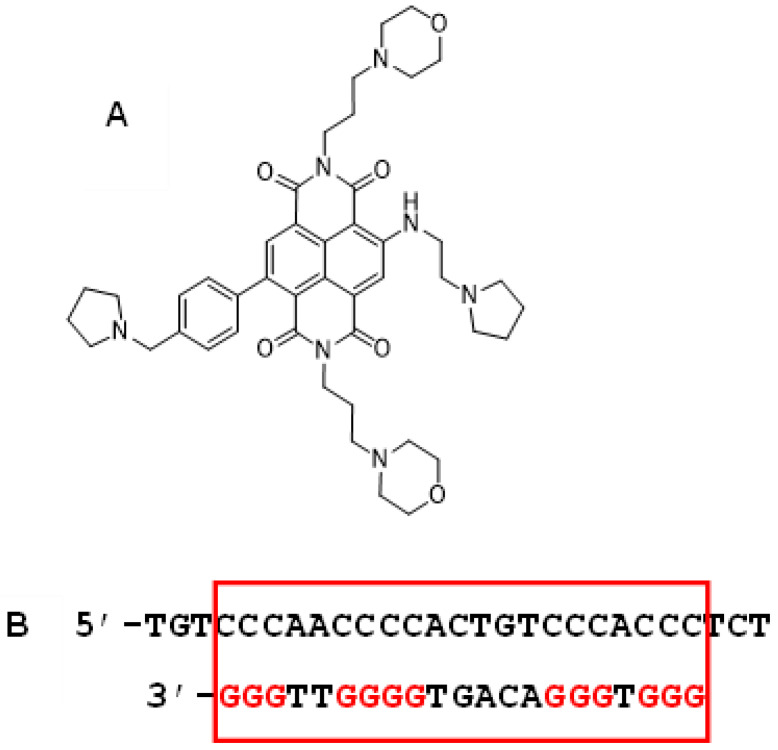
(**A**) Structure of QN-302 (2,7-bis(3-morpholinopropyl)-4-((2-(pyrrolidin-1-yl)ethyl)amino)-9-(4-(pyrrolidin-1-ylmethyl)phenyl)benzo [lmn] [3,8]phenanthroline-1,3,6,8(2H,7H)-tetraone). (**B**) DNA sequence in the promoter region of the S100P gene found to form a G-quadruplex. The G4 sequence itself is bounded within the red box and the individual G-tracts are highlighted in red.

We have adopted an approach to the development of an effective small-molecule therapy for PDAC, based on targeting the prevalence of discrete “signal” quadruplex sequences within cancer genes, as described below. The compound QN-302 (Figure 1A), a tetra-substituted naphthalene diimide derivative, has previously been disclosed [21] to have single-digit nM anti-proliferative activity in a panel of human pancreatic ductal adenocarcinoma (PDAC) cell lines and significant anti-tumor activity in the MIA PaCa-2 xenograft model for PDAC, with a 91% reduction in tumor volume relative to the vehicle control arm (*p* = 0.008) for twice-weekly dosing over a four-week period. A statistically significant increase in survival for treated animals (*p* = 0.016) was also observed in the KPC genetically engineered mouse model for PDAC. QN-302 has good bioavailability at therapeutic doses and is currently in advanced pre-clinical development with Qualigen Therapeutics Inc. It has also recently (Jan 2023) been granted Orphan Drug Designation status by the FDA in the USA for the treatment of pancreatic cancer.

The proposed mode of action of QN-302 involves high-affinity (ca 1 nM) binding to and stabilization of G-quadruplex (G4)-forming sequences [22]. G4s are formed by the folding into higher-order structures of short repetitive DNA and RNA guanine-rich sequences [23,24,25]. These are over-represented in the promoter regions of many cancer-related and proliferative genes [26,27,28,29,30]. This stabilization is believed to inhibit transcription factor binding and the progression of RNA polymerase and thus directly results in downregulation of gene expression at the transcriptional level. Transcriptome (RNA-seq) analyses of the effects of QN-302 [21] and the related compound CM03 [31] in MIA PaCa-2 cells has confirmed this hypothesis and has revealed a pattern of susceptible genes involved in cancer-associated pathways. This has also confirmed that the downregulated genes are over-represented with G4 sequences in their promoters. Notable G4-containing genes downregulated by QN-302 are in the mTOR, axon guidance, VEGF, insulin and Wnt/β-catenin pathways, which are implicated in PDAC disease and progression. The major gene changes in gemcitabine-treated cells have also been mapped and shown to generally not affect these G4-containing genes sensitive to QN-302 [21,32]. Consequently, PDAC cells with induced gemcitabine resistance retain their sensitivity to these G4 ligands.

We report here on a further analysis of the transcriptomic data in PDAC cells, which revealed that the *S100P* gene is prominent among the most downregulated by QN-302 treatment. Other in vitro and in vivo studies described here have revealed it to be a gene that is knocked down by QN-302 not only in PDAC cells but also in a PDAC xenograft model. The expression of this gene, which codes for a small (10.4 kDa) calcium-binding protein [33,34], has been found to be highly upregulated in 70.4% of a cohort of 176 human PDAC patients [35], and correlates with disease status. S100P induces MAPK/ERK as well as PI3K/AKT growth-promoting pathways and S100P knock-out leads to P53-mediated cancer cell death. It has been proposed as a plausible biomarker for diagnostic purposes and possibly also as a therapeutic target in PDAC, as well as in colorectal cancer [33,34,36,37,38,39]. Thus, the central question addressed in this study has been whether QN-302 treatment in cells and in vivo have effects on the *S100P* gene and its expressed protein that supports it being suitable as a prognostic biomarker for this drug, although the mechanistic details are yet to be fully established.

## 2. Results

### 2.1. RNA-seq Analysis of RNA from Cell-Based Studies

RNA-seq analysis of the effects of 24 h exposure of QN-302 on MIA PaCa-2 cells have previously shown that the mRNA levels in the prominent cancer pathway quadruplex-containing genes such as *GLI1*, *MAPK11* and *BCL*-2 were downregulated by 1–2 fold [21]. Table 1 lists several other PDAC-related genes selected from the list of 229 genes in the cancer genetics web site [40] as also having significantly downregulated expression in this RNA-seq data set. Genes *S100P* and *CX3CL1* are the most prominent in this set, and the former was selected for further study in view of its well-established upregulation role in PDAC (see below), which was also supported by the patient-derived transcriptome data presented in Table 1 and below. The downregulation of *S100P* expression, by 3.23 log_2_ fold (89.8% downregulation relative to controls), is within the top 0.1% cohort of gene changes in the complete MIA PaCa-2 transcriptome.

### 2.2. QN-302 Downregulates S100P Expression in an In Vivo Xenograft Model

**Figure 2:**
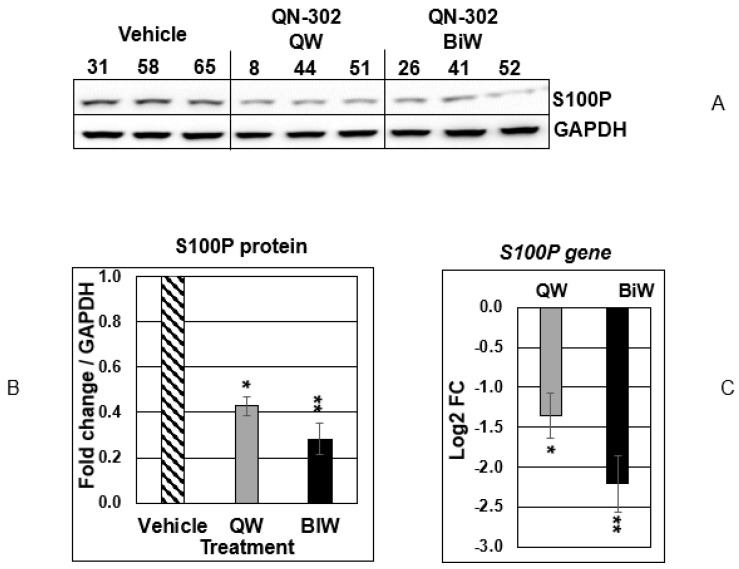
(**A**) Western blots of S100P protein and GAPDH control, for protein extracted from the results of the xenograft experiment with MIA PaCa-2 implanted tumors and a vehicle control arm, at day 28 of the study. The number at the top of each individual column represents an individual mouse. QW: once-weekly dosing. BiW: twice-weekly dosing. (**B**) Quantitation of the Western blot data. Statistical significances are indicated by * *p* < 0.05, ** *p* < 0.01. (**C**) Quantitation of the qPCR data for the *S100P* gene in the treated vs. vehicle control animals. Statistical significances are indicated by * *p* < 0.05, ** *p* < 0.01 (one-way ANOVA test).

Figure 2A–C show the results of quantitation of protein and mRNA expression for S100P and its gene product for weekly and bi-weekly dosing using the MIA PaCa-2 xenograft model. In each case, as for the vehicle control arm, data are available for three mice. S100P protein expression was downregulated by 60% (*p* < 0.05) for weekly dosing and by 75% (*p* < 0.01) for twice weekly (Figure 2A,B), implying a dose-dependent effect. Similar reductions in mRNA levels were also observed (Figure 2C).

### 2.3. RNA-seq Analysis of Human PDAC Tumor Tissues

To identify direct PDAC-related targets and/or potential biomarkers for QN-302, RNA-seq was performed on a set of four PDAC patient samples: two from male patients diagnosed with poorly differentiated adenocarcinoma with invasion and two from male patients diagnosed with moderately differentiated ductal adenocarcinoma with invasion. Table 2 shows a list of PDAC patient samples with their diagnoses, obtained from University of Liverpool, four of which were chosen for RNA-seq. Normal pancreatic samples of three healthy male individuals with age-matching the PDAC patients were obtained commercially from OriGene and used in the differential gene expression analysis (Table 2). The full RNA-seq data sets determined in this analysis are shown in Table S1.

**Figure 3:**
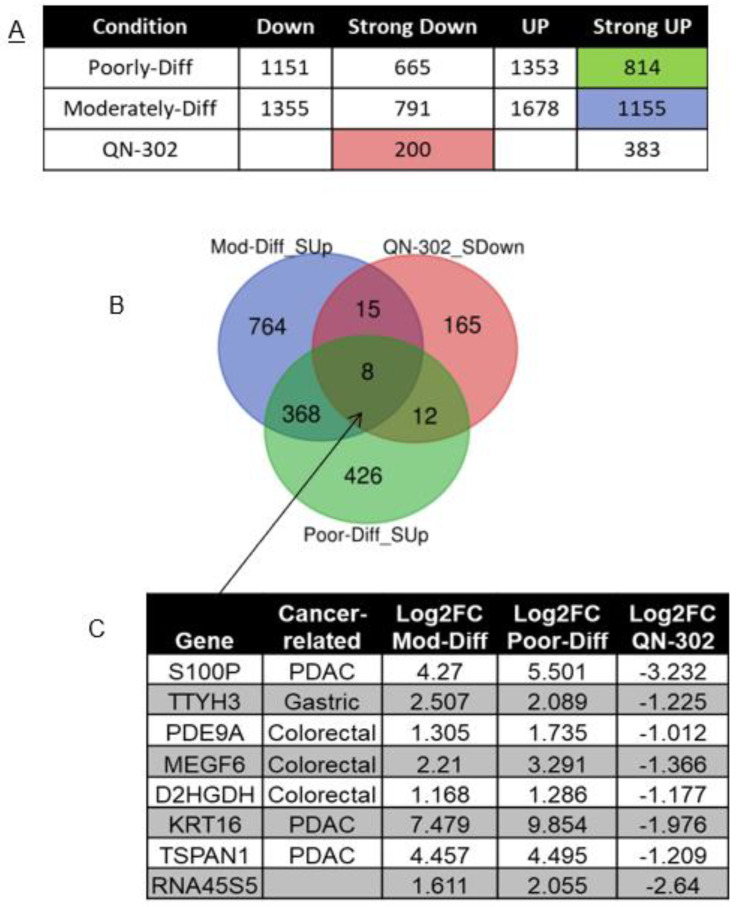
(**A**) Numbers of differentially expressed genes (DEGs) from RNA-seq of PDAC patient samples (poorly or moderately differentiated) versus normal pancreas tissue. DEGs from QN-302 study were added to compare with PDAC data. DEGs were split into four subsets: Down = DEGs with log_2_FC < −0.5 and FDR < 0.1; Strong Down = DEGs with log_2_FC ≤ −1 and FDR < 0.05; Up = DEGs with log_2_FC > 0.5 and FDR < 0.1; Strong Up = DEGs with log_2_FC ≥ 1 and FDR < 0.05. (**B**) Venn plot showing numbers of upregulated genes in PDAC patients intersected with genes downregulated by QN-302 when treating PDAC MIA PaCa-2 cells for 24h. (**C**) List of the common upregulated genes in poorly and moderately differentiated PDAC patients, potential targets for QN-302.

Figure 3A shows the numbers of differentially expressed genes (DEGs) in poorly and moderately differentiated PDAC, divided into four subsets as indicated. To identify the upregulated genes in PDAC which could be targeted by QN-302, DEGs with log_2_FC ≥ 1 and FDR < 0.05 (Strong UP) in poorly and moderately differentiated PDAC were intersected with DEGs with log_2_FC ≤ −1 and FDR < 0.05 (Strong Down) in the QN-302-dosed cell data. The Venn diagram shows the shared and unshared number of genes between the three conditions (Figure 3B). There are eight genes common for all three conditions, which are involved in PDAC and/or other cancers. The genes *TSPAN1*, *KRT16* and *S100P* are highly upregulated in PDAC and downregulated by QN-302, all of which could be considered as potential therapeutic biomarkers. Since S100P has previously been extensively studied as a potential biomarker in PDAC diagnosis (see Discussion), we focused on it in this study, while the other genes may be considered in a future study.

**Figure 4:**
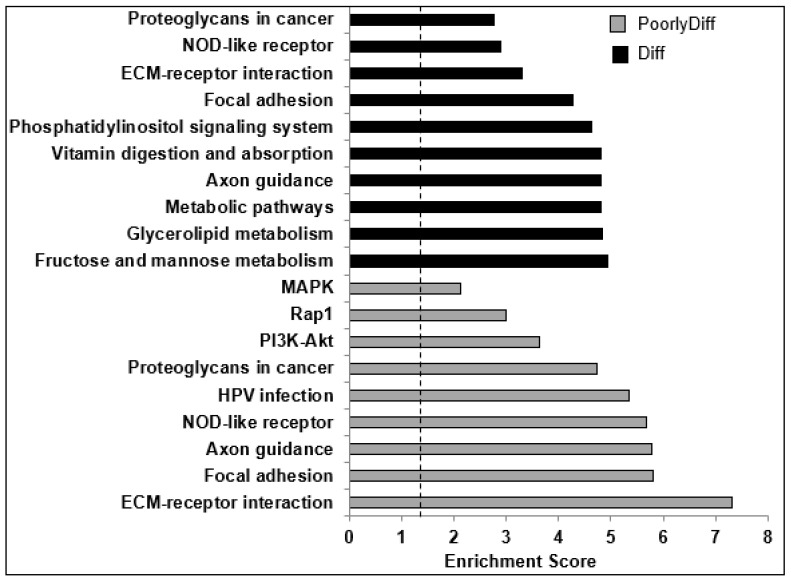
Graph of significantly enriched KEGG pathways (p-EASE ≤ 0.05) of upregulated genes (UP subset = Log_2_FC > 0.5, FDR < 0.1) in both poorly and moderately differentiated PDAC patient samples when compared to normal pancreatic tissue. Above the dotted line represents statistical significance at *p* value  <  0.05, as calculated by Fisher’s exact test.

KEGG pathways enrichment analysis shows the similar top-affected signaling pathways between poorly and moderately differentiated PDAC, such as ECM–receptor interaction, focal adhesion, and axon guidance (Figure 4 and Table S1). Interestingly, two of these signaling pathways are also targeted for downregulation by QN-302 [21]: 1. the axon guidance pathway common to both PDAC stages; and 2. the Rap1 pathway, in poorly differentiated PDAC.

### 2.4. Bioinformatics Analyses

The QGRS Mapper program located 28 putative quadruplex sequences in the coding strand and 32 in the template strand. The majority of these have low G scores, i.e., have reduced likelihood of forming stable quadruplexes. However, of these a total of six have plausible stable G4 sequences (Table 3). All bar one occurs in intronic regions. The exception occurs within the S100P promoter, 48 nucleotides upstream from the transcription start site. The same sequence also occurs in the highly homologous mouse S100P gene (ENSG00000163993). This putative quadruplex sequence on the template strand, and its C-rich complement on the coding strand, were used for subsequent biophysical evaluations.

### 2.5. Biophysical Studies

**Figure 5:**
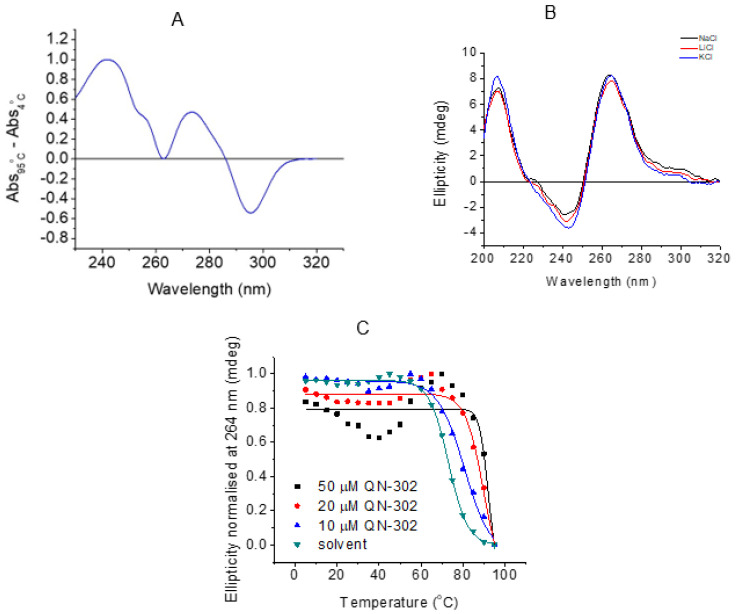
(**A**) Thermal difference spectra of 5 µM S100P PQS in 10 mM lithium cacodylate, 100 mM KCl buffer at pH 7.0. (**B**) CD spectra of 10 µM S100P quadruplex sequence (Figure 1B) in 10 mM lithium cacodylate, pH 7.0 and 100 mM of either KCl, NaCl or LiCl, as indicated. (**C**) Representative CD melting experiments with10 µM S100P quadruplex in 10 mM lithium cacodylate, pH 7.0, 100 mM KCl and 0, 10, 20 or 50 µM QN-302 as indicated.

All experiments reported here were performed with the putative G-quadruplex promoter sequence from S100P, as detailed above. UV thermal difference spectroscopy was initially performed to characterize the structure formed in 10 mM lithium cacodylate, 100 mM potassium chloride buffer at pH 7.0 (Figure 5A). A TDS with positive peaks at 240, 275 nm and a negative peak at 295 nm is consistent with a G-quadruplex structure [41]. CD spectroscopy in the presence of either 100 mM KCl, NaCl or LiCl gave spectra of the same general form (Figure 5B), with positive bands at 210 and 265 nm, and a negative band at 245 nm, are consistent with a predominantly parallel G-quadruplex structure [42], a shoulder at 295 nm also indicates a small proportion that is antiparallel. CD melting experiments of S100P in buffer containing 10 mM lithium cacodylate and 100 mM KCl buffer at pH 7.0 gave a transition at 73°C, indicating the G-quadruplex structure formed would be highly stable under physiological conditions. Adding 10 μM (1 eq) of QN-302 to the S100P G4 resulted in a ΔT_m_ of 7.4 ± 0.2 °C, and 20 μM (2 eq) and 50 μM (5 eq) had correspondingly higher ΔT_m_ values of 17.0 ± 0.1 and 20.0 ± 1.3 °C, respectively, where at 50 μM the melting was at the limit of what can be accurately measured under these experimental conditions (Figure 5C). The largest increase in melting temperature was achieved with 2:1 ligand:DNA stoichiometry and there was not much further increase in melting temperature on addition of five equivalents of ligand. These data (Figindicate that QN-302 has a strong stabilising effect on the G-quadruplex structure formed.

### 2.6. Molecular Modeling

**Figure 6:**
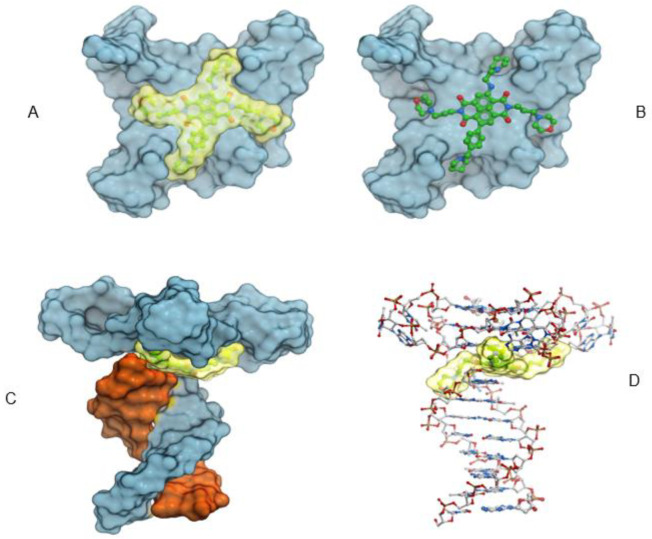
Four views of the docked model of QN-302 with a quadruplex–duplex model structure. In (**A**–**C**) the quadruplex-forming strand is colored grey and the complementary duplex stem shown in (**C**) is colored orange. (**A**,**B**) were drawn viewing the G-quartet and QN-302 plane and show the four substituents each in a quadruplex groove. In each instance the quadruplex is drawn as a grey solvent-accessible surface. A shows the QN-302 molecule as a yellow solvent-accessible surface, and in (**B**) it is shown in ball-and-stick mode. In (**C**,**D**) the complete quadruplex-duplex model is shown together with bound QN-302 in solvent accessible representation and colored yellow. (**D**) (with the DNA shown in stick mode) shows that one of the morpholino groups OF QN-302 has interactions in a groove at the junction of the G4-duplex DNA (with an N3 atom).

Docking studies onto the parallel G-quadruplex determined that the core of the QN-302 molecule lies in an energetically favorable position at the center of the terminal G-quartets and is positioned directly above the central electronegative channel of the quadruplex. The QN-302 side chains access the four grooves and the protonated nitrogen atoms in the morpholino and the pyrrolidine side chains have electrostatic interactions with the phosphate backbone or the N3 atoms in the guanines within the top quartet. The benzene ring in the benzyl-pyrrolidine side chain acts as an extension of the planar naphthalene diimide chromophore core and makes π stacking interactions with the imidazole ring in the guanines. Very similar interactions are also observed in the generated complex between QN-302 and the G4-duplex junction (Figure 6A–D). The only difference between the pure quadruplex and the junction structure is that in the latter one of the morpholino groups has interactions with the N3 atom from the duplex DNA nucleotide at the junction. It is notable that QN-302 exploits the same interactions at the 3′ terminal quartet (as in the telomeric G4) as well as on the 5′ end at the G4-duplex junction.

## 3. Discussion

The results presented here show that expression of the *S100P* gene at the mRNA and protein levels in a PDAC cell line and in a PDAC xenograft model is highly downregulated by the quadruplex-binding compound QN-302. We also show that *S100P* mRNA is over-expressed in tumors from human PDAC patients. This is consistent with the concept that the extent of enhanced expression correlates with disease progression (and prognosis) for moderately and poorly differentiated PDAC human tumors, albeit for the small tumor sample size in the present study. This result is in accord with numerous other studies [33,34,35,36,37,38,39] and reflects the role that appears to be played by S100P in PDAC, where it promotes proliferation, tumorigenesis, invasion and progression [33,34]. We conclude that the data support previous studies (for example, refs [36,37,38,39]) showing that S100P is a viable biomarker for PDAC. Monitoring of S100P levels may also be a useful predictor of tumor response to treatment with the experimental drug QN-302. The potential advantages of S100P as a biomarker in PDAC diagnosis have been extensively documented [33,34,35,36,37,39], comparing favorably to CA19-9 antigen, which can produce both false positive and false negative indications of disease [44,45].

Evidence is also presented that the putative quadruplex sequence in the promoter region of the *S100P* gene [46] folds into a highly stable G-quadruplex in solution conditions of physiological potassium ion concentration. Circular dichroism analysis indicates that the quadruplex has a parallel topology that is retained on binding QN-302. This compound imparts further stability to this quadruplex, as shown by an increase in melting temperature. We suggest that the molecular model presented here is representative of a more generalized parallel quadruplex ligand complex structure, in that the similarity in the mode of QN-302 binding to that found in naphthalene diimide quadruplex complex crystal structures [47,48] gives plausibility to the model, even in the absence of detailed experimental structural data on this S100P quadruplex or its QN-302 complex. It is notable that the modelled geometry of the duplex–quadruplex hybrid complex [49] is remarkably close to that recently reported by NMR methods [50]. The S100P quadruplex sequence contains three putative loop regions, of which one, comprising the single nucleotide T has the most frequent occurrence in quadruplex loops [51], where its geometry is normally only consistent with a parallel quadruplex topology. The biophysical data is thus consistent with (but does not prove) the hypothesis that these changes in *S100P* expression are a consequence of QN-302 binding to and stabilizing this promoter G-quadruplex. Definitive proof of a direct causal relationship as compared to correlative evidence must await further studies. There are many reports in the literature of analogous downregulatory effects on the transcription of other promoter quadruplex-containing genes that have been ascribed to quadruplex binding, notably to the c-*MYC* [52,53], h-*TERT* [54,55] and c-*KIT* [56,57] oncogenes. Transcriptional suppression by QN-302-induced G-quadruplex stabilization may, in part, be due to physical blocking of the movement of RNA polymerase along the template strand, and in part to competition with normal transcription factor binding at this promoter site, as has been demonstrated for the ligand pyridostatin [58]. The *S100P* promoter quadruplex box is known to be a binding site for the SP/KLF transcription factors [46].

Small molecule [59] and antibody [60] approaches successfully targeting S100P have previously been reported. For example, S100P knockdown experiments by siRNA resulted in apoptosis in endometrial epithelial cells [61]. It is hoped that future studies, beyond the scope of this manuscript, will establish the causal basis of the relationship between QN-302 binding to the S100P promoter G4 and in vitro/in vivo effects. Elevated S100P expression has been found in several other cancers, including colorectal [38], lung [62,63], where it correlates with the activity of the oncogenic PI3K/AKT signaling pathway, and gall bladder cancers [64]. Evidence for S100P being a viable anticancer target is also provided by an aptamer approach in colorectal cancer with high affinity to and selectivity for S100P protein. This aptamer has shown high activity in cells and in a xenograft model for this disease [65].

## 4. Materials and Methods

QN-302 (>98% purity, as judged by LCMS) was used as the free base. Its synthesis and purification have been previously described [21].

### 4.1. RNA-seq Analysis of RNA from Cell-Based Studies

These have been previously reported in detail [21], and the process of determining changes in transcription upon exposure of MIA PaCa-2 cells to QN-302 has been fully described. The RNA-seq data is available in the GEO public functional genomics data repository, as GSE151741 (https://www.ncbi.nlm.nih.gov/geo/), accessed on 12 June 2022.

### 4.2. RNA-seq Analysis of Tumor Material from Xenograft Studies

This employed the MIA PaCa-2 xenograft model for PDAC. All animal experiments in this section were performed at AXIS BIO Discovery Services Northern Ireland, in accordance with the UK Home Office Animals Scientific Procedures Act 1986 and the United Kingdom Co-ordinating Committee on Cancer Research Guidelines for the Welfare and Use of Animals in Cancer Research^4^, and with the approval of the AXISBIO Animal Ethics Committee. Mice had access to food and water ad libitum.

For therapy studies [21], female athymic nude mice (2–3 months old, weighing 20−25 g) were injected subcutaneously with 10^7^ MIA PaCa-2 cells in Matrigel in the right flank. When the tumors were established (approximately 13 days, mean size 0.05 cm^3^), the mice were randomly assigned into treatment groups with eight mice per group. Compound QN-302 was administered IV, in sterile PBS (pH 6), plus a few drops of 0.1 mM HCl if needed to ensure complete solubilization, on a twice weekly basis, for 28 days. The vehicle control used was saline only, also with twice-weekly dosing. Tumor size was measured 3 times weekly by caliper using the π-based ellipsoid volume formula (length × width × height × π/6), and the mice were also weighed at the same time. Animals were examined daily for any signs of distress or toxicity from the treatments. Results of the therapy experiments are detailed elsewhere [21].

Quantification of expressed S100P protein and mRNA within tumor tissue was undertaken, using tissue taken from three control and three treated animals sacrificed at day 28 of the xenograft experiment.

Western blotting. Frozen tumor tissue was thawed and lysed in RIPA (radio-immunoprecipitation assay) buffer (CST#9806) supplemented with phosphatase and protease inhibitors (CST#5872) and PMSF protease inhibitor (CST#8553). Lysate was isolated and total protein quantified for each sample using a commercially available assay kit. 30 µg total protein was loaded onto a 4–20% polyacrylamide gel and electrophoresed. Separated protein was transferred to a PVDF membrane and blocked in 5% BSA. Primary antibodies as listed below were diluted 1:1000 in 5% BSA and incubated at 4°C overnight with constant rolling. Membranes were washed in TBS-T (a mixture of tris-buffered saline and polysorbate 20) before being incubated with secondary antibody (diluted 1:3000 in 5% BSA) at room temperature for 1h. Following washes in TBS-T, bands were observed following addition of SignalFire Plus ECL reagent (CST#12630). Images were taken using a GeneGnome imaging system (Syngene, UK). Densiometric analysis of the bands was carried out using Genesys image analysis software (part of the GeneGnome system). Statistical significances were analyzed using a one-way ANOVA test with a Bonferroni correction, where * *p* < 0.05 and ** *p* < 0.01.

Antibodies:
**Target****Supplier****Catalogue Number****Dilution****Incubation Details**S100PCell Signaling Technology76771:1000Overnight @4 °CGAPDHCell Signaling Technology51741:1000Overnight @4 °CAnti-Rabbit IgGCell Signaling Technology70741:3000RT for 1 h

*qPCR.* RNA was isolated from frozen tumor tissue using a GeneJET RNA isolation kit (Thermo Fisher; K0731). Isolated RNA was subjected to a 1-step RT-qPCR process using a Superscript III Platinum kit (Thermo Fisher, Waltham, MA, USA; 11736051). Reactions were set up in 96-well format in the LightCycler 480 apparatus and run according to the manufacturer’s instructions. Primer sequences used are listed below. HPRT (hypoxanthine-guanine phosphoribosyltransferase) was included as a reference gene. Reactions for every gene were set up in triplicate, including non-template controls. For analysis, we used CT (cycle threshold), and ΔCT, ΔΔCT, and RQ (relative expression) values were calculated according to the 2^-ΔΔCT^ method. Statistical significances were analyzed using Student’s t test (GraphPad Inc., San Diego, CA, USA), where * *p* < 0.05 and ** *p* < 0.01.
**Target Gene****Forward Primer****Reverse Primer**S100PCTCAAGGTGCTGATGGAGAAGGGAACTCACTGAAGTCCACCTGGHPRT1CATTATGCTGAGGATTTGGAAAGGCTTGAGCACACAGAGGGCTACA

### 4.3. RNA-seq Analysis of Human PDAC Tumor Tissues

Transcriptome analyses were performed on a supplied panel of RNA samples, comprising RNA from a set of poorly differentiated as well as from more highly differentiated human pancreatic tumors, and the results have been compared with normal pancreas expression data. The RNA-seq data are available in the GEO public functional genomics data repository (https://www.ncbi.nlm.nih.gov/geo/), as accessions GSE226307 and GSM7071068-GSM7071074, deposited on 28 February 2023.

The normal pancreas samples were obtained commercially from OriGene Technologies GmbH Germany (Cat #: CR560569, CR562915, CR561640) and the averaged gene expression data of the three samples were used in the analysis. The PDAC RNA samples were obtained from pancreatic tumor tissue with the Maxwell^®^ RSC SimplyRNA Tissue Kit (Promega Ltd. Loughborough, UK, Cat # AS1340). In brief, the tissue was snap frozen in the operating theatre following tumor removal and then homogenized on chilled 1-thioglycerol before adding 200 µL to a Maxwell RSC Cartridge and running on a Maxwell RSC instrument according to the manufacturers’ instructions. The samples were obtained with ethical approval from the NRES Committee North-West–Liverpool Central, MREC 07/H1005/87.

RNA quality (RIN > 7.0) was checked with an Aligent 2100 Bioanalyser RNA 6000 Nano Chip and RNA concentration was quantified using a Qubit^®^ fluorometer (ThermoFisher;Waltham, MA, USA ) and Qubit^®^ RNA HS Assay Kit (ThermoFisher, cat #: Q32852). RNA-seq libraries were then generated using the KAPA mRNA Library HyperPrep Kit for Illumina^®^ following the manufacturer’s instructions and sequenced using an Illumina NextSeq 500 instrument (undertaken at the UCL Genomics Facility).

The sequence data were demultiplexed and converted to FASTQ files using Illumina’s bcl2fastq Conversion Software (v2.19). The adapter contamination and poor-quality sequences were removed from FASTQ files using the program Trimmomatic (v0.36) [66]. FASTQ files were then mapped to the human reference genome GRCh38 using the RNA-seq aligner STAR (v2.5b: https://github.com/alexdobin/STAR), accessed on 12 February 2020. The JE-Suite procedure [67] was used to estimate duplication levels, using a Unique Molecule Identifier program to deduplicate, and then mark the reads that are the result of PCR amplification. Then, reads per transcript were counted using the program FeatureCounts [68] (v1.4.6p5) followed by normalization, modeling and differential expression analysis using the SARTools (v1.3.2) package [69], accessed on 14 February 2020.

Differentially expressed genes (DEGs) for PDAC versus normal pancreas were split into four subsets with defined log_2_ fold change (log_2_FC) and false discovery rate (FDR) with the following assignment: Down = DEGs with log_2_FC < −0.5 and FDR < 0.1; Strong Down = DEGs with log_2_FC ≤ −1 and FDR < 0.05; Up = DEGs with log_2_FC > 0.5 and FDR < 0.1; Strong Up = DEGs with log_2_FC ≥ 1 and FDR < 0.05. The KEGG signaling pathway enrichment analysis was performed using the DAVID functional annotation tool [70] (https://david.ncifcrf.gov/), accessed on 14 February 2020.

### 4.4. Bioinformatics Analyses

The DNA sequence of the human S100P gene was extracted from the ENSEMBL genome browser, release 108 (https://www.ensembl.org/, accessed on 12 June 2022), as entry noENSG00000163993. The complete gene sequence contains 4493 nucleotides. The program QGRS Mapper [45] (https://bioinformatics.ramapo.edu/QGRS/index.php), accessed on 12 June 2022 was used to locate putative quadruplex sequences in both sense (coding) and antisense (template) strands of the complete S100P gene. A cut-off of a maximum loop size of 12 nucleotides was used in the searches.

### 4.5. Biophysical Studies

The putative G-quadruplex-forming sequence (PSQ) from the S100P promoter, as found from the bioinformatics analyses, 5′-d[AGGGTGGGACAGTGGGGTTGGGA]-3′, was purchased from Eurogentec UK and was supplied rpHPLC purified and as a dry solid. The DNA was initially dissolved as a stock solution in purified water (1.77 mM); further dilutions were carried out in the respective buffer. Samples were thermally annealed in a heat block at 95 °C for 5 minutes and cooled slowly to room temperature overnight. A stock solution of QN-302 was prepared in buffer, with some drops of 0.1 M HCl to aid solubilization. Data were analyzed using the OriginLab package (https://www.originlab.com/).

Thermal Difference spectra (TDS) were performed on a Jasco V-750 UV–Vis spectrometer. The oligonucleotide sample was diluted to 5 µM in 10 mM lithium cacodylate, 100 mM KCl buffer at pH 7. After annealing, the sample (250 μL) was transferred to a quartz 10 mm cuvette and stoppered to reduce evaporation. The absorbance at wavelengths between 230 and 320 nm was measured for the folded structure at 4 °C and the unfolded structure at 95 °C. The sample was equilibrated for 5 to 10 min at each of the two temperatures before recording the absorbance. To calculate the TDS, the spectrum of the folded structure was subtracted from the unfolded structure spectrum. The resulting spectrum was zero corrected at 320 nm. Data was analyzed using OriginLab.

Circular Dichroism (CD) experiments were recorded on a Jasco J-1500 spectropolarimeter using a 1 mm path length quartz cuvette. To characterize and examine the effect of different cations on the S100P sequence, CD spectra were recorded in the presence of Na^+^, Li^+^ and K^+^ cations. In each case, the 10 μM DNA sample was thermally annealed in 10 mM lithium cacodylate with either 100 mM of NaCl, LiCl and KCl, respectively at pH 7 (total volume: 100 μL). The scans were recorded at room temperature between 200 and 320 nm. Data pitch was set to 0.5 nm and measurements were taken at a scanning speed of 200 nm/min, data integration time, of 1 s, bandwidth of 1 nm. Each spectrum was the average of four scans. Samples containing only buffer were also scanned to allow for blank subtraction. The spectrum was zero corrected at 320 nm.

Circular dichroism spectroscopy was used to measure any ligand-induced effects on the stability of the DNA structure. DNA samples were thermally annealed in 10 mM lithium cacodylate, 100 mM KCl pH 7 at 10 μM using 100 µL per sample. Melting experiments were performed in the presence and absence of 1 and 5 ligand equivalents (10 μM and 50 μM QN-302) while heating the sample at a rate of 1 °C/min from 5 to 95 °C and measuring at 5 °C intervals. The temperature at which 50% of the thermal denaturation had taken place (T_m_) was calculated by using OriginLab data analysis software to plot normalized ellipticity against temperature. These data were fitted with sigmoidal dose–response curves to give the T_m_ values. Final values are given as the average and standard deviation of two repeats.

### 4.6. Molecular Modeling

The crystal structures of the parallel stranded quadruplex [71] formed from the human telomeric sequence and its complex with the earlier-generation naphthalene diimide compound MM41 [47] (PDB entry 3UYH) were used as models to assess how QN-302 interacts with parallel-topology quadruplexes in general. To generate QN-302, the piperazine side chains in MM41 were replaced with propyl-pyrrolidine and benzyl-pyrrolidine, while retaining the morpholino side chains. The coordinates of QN-302 were used to generate a grid, which extended 10 Å around the ligand and encompassed the terminal quartet and the loops of the quadruplex structure. QN-302 was docked using MolSoft ICM 3.9-3a software (https://www.molsoft.com/). A similar protocol was adopted to dock QN-302 at the junction of a previously generated model for a parallel quadruplex-duplex DNA hybrid [49].

## Figures and Tables

**Table 1:** Expression changes in selected genes previously identified as human PDAC-associated and responsive in QN-302 cell studies. Gene data taken from list of 229 genes with mutations and/or altered expression in PDAC, detailed in the Cancer Genetics Web site: http://www.cancerindex.org/geneweb/X0603.htm, accessed on 17 November 2022. mRNA data were taken from UCL RNA-seq transcriptome data on MIA PaCa-2 cells dosed with QN-302 [21]. PSQs are the number of putative quadruplexes found in a gene sequence.

Gene	QN-302, 24 h in MIA PaCa-2 Cells	*p* Value	PDAC P-D Minus Normal	*p* Value	No of PGQs	Gene Function	PDAC Occurrence and Function
SPARC	−2.252	0.61	2.685	0.071	10	Secreted protein acidic and cysteine rich	Promotes pancreatic cancer cell proliferation and migration
**S100P**	**−3.230**	**0.08**	**45.27**	**0.054**	**60**	**Calcium binding protein involved in multiple signal transduction pathways**	**Sensitive and specific marker for the detection of PDAC, promotes PDAC growth and survival**
ID2	−1.215	0.0001	0.398	0.014	3	DNA-binding pro-proliferative gene	Over-expressed in PDAC
CLDN7	−2.39	0.429	1.213	0.643	4	Involved in junction formation	Over-expressed in PDAC and other cancers
CX3CL1	−2.912	0.040	2.87	0.017	5	Chemokine	Modulates the development of PDAC via JAK/STAT signalling pathway; upregulated in PDAC

**Table 2:** Data on patient samples of RNA taken from human PDAC tumors and the normal pancreas samples used in this study.

Sample ID	Age	Gender	Diagnosis/Tissue Composition
R2971	65	Male	Poorly differentiated adenocarcinoma with invasion (PanIN)
R2944	65	Male	Poorly differentiated ductal adenocarcinoma with necrosis (PanIN)
R2824	71	Male	Moderately differentiated ductal adenocarcinoma of the head of pancreas with invasion (ductal epithelial changes in high grade PanIN)
R2700	70	Male	Moderately differentiated ductal adenocarcinoma with invasion and necrosis (PanIN)
CR560569	53	Male	Normal pancreatic tissue comprised of 75% Exocrine epithelium, 10% Endocrine epithelium, 15% Ducts
CR562915	66	Male	Normal pancreatic tissue comprised of 90% Acini, 5% Ducts, 5% Islet cells
CR561640	69	Male	Normal pancreatic tissue comprised of 90% acini, 0% islets, 5% ducts, 5% fibrofatty stroma

**Table 3:** Top predicted quadruplex sequences in the S100P gene, as found using the QGRS Mapper program (https://bioinformatics.ramapo.edu/QGRS/index.php), accessed on 12 June 2022. The G4 score, as defined in [43], is a measure of the likelihood of the sequence forming a stable quadruplex under physiological conditions. Only those sequences with a G-score >35 are listed here. G-tracts are underlined. The highlighted sequence, in the S100P promoter, is discussed further below.

Position in Seq	5′-Sequence	G4 Score	Strand	Gene Region
2251	**GGGAACTTGGGCTTGGGGCTCGGGG**	41	Coding	Intron
2524	**GGGCAGGGCTGGGCTGGG**	42	Coding	Intron
548	**GGGTGGGACAGTGGGGTTGGG**	38	Template	Promoter
1239	**GGGAGCAGGGAAAGCGGGCAAGTGGG**	41	Template	Intron
1487	**GGGGTCAAAGGGAAGGGGAAATGGAGAGGG**	36	Template	Intron
2467	**GGGAGGGCTGAGAAGGGCACCCAGGG**	36	Template	Intron

## Data Availability

Transcriptome data has been deposited and is available from the GEO public functional genomics data repository (https://www.ncbi.nlm.nih.gov/geo/), as GSE151741 (cell-based studies) and GSE226507, GSM7071068-1074 (for patient-derived material). Details of the molecular models are available from the authors on reasonable request.

## Supplementary Materials

The following supporting information can be downloaded at: https://www.mdpi.com/article/10.3390/molecules28062452/s1, Table S1 comprising RNA-seq data for expression of the genes from human PDAC compared to those from normal pancreas.
